# Prostate-specific *PTen* deletion in mice activates inflammatory microRNA expression pathways in the epithelium early in hyperplasia development

**DOI:** 10.1038/s41389-017-0007-5

**Published:** 2017-12-14

**Authors:** D. Alwyn Dart, Pinar Uysal-Onganer, W. G. Jiang

**Affiliations:** 1 Cardiff China Medical Research Collaborative, Cardiff University School of Medicine, Henry Wellcome Building, Heath Park, Cardiff, CF14 4XN UK; 2 Dept. of Biomedical Sciences, Faculty of Science and Technology, University of Westminster, 115 New Cavendish Street, London, W1W 6UW UK

## Abstract

PTen loss is one of the most frequent events in prostate cancer both at the initiation stage and during late stage metastatic development. The mouse model of prostate-specific probasin-mediated *Pten* deletion leads to prostate intraepithelial neoplasia (PIN) leading to adenocarcinoma. Using this model, we analysed the miR and mRNA transcriptome profile of *Pten* ^−/−^ PIN versus wild type age-matched prostate tissues and analysed the effects of *Pten* loss on miR expression in the early neoplastic process. At the PIN stage, *Pten* loss significantly changed the expression of over 20 miRNAs and over 4000 genes. The observed miR expression indicated a strong immunological cohort, which is seen in many human and mouse cancers and is thought to derive from infiltrating B and T immune cells. However, upon in situ hybridisation, these immunologically related miRs did not correlate with immune cell location, and emanated from the prostate epithelium itself and not from the associated immune cells present. Growing *Pten* ^−/−^ prostate cells in culture showed that the overexpressed miRNAs seen in *Pten* ^−/−^ were directly in response to the overactive PI3 kinase pathway and were in part responsible in reducing target gene expression levels. Inhibition of PI3 kinase downstream regulators, or re-introducing wild type *Pten* ^cDNA^ reduced miR overexpression resulting in increased miR target gene expression. MiR inhibitors also showed this pattern, and synergised with an mTORC1 inhibitor. Overall, *Pten* deletion in the prostate epithelium activated a cohort of inflammation-related miRs usually associated with immune responses from B and T cells. These oncomiRs may then accelerate carcinogenesis.

## Introduction

MicroRNAs (miRs) are involved in many diseases, including prostate cancer (PCa), and are being developed as predictive and prognostic markers of disease, or indeed as therapies. miRs are small RNAs that associate with 3′ untranslated regions of specific mRNAs causing degradation or translational inhibition^1,2^. Approximately 30% of genes are thought to be controlled by miRs^3^. MiRs can regulate several mRNAs and each mRNA is regulated by several miRs, leading to complex interactions. MiRs regulate diverse biological events, from cell division, morphology to tissue development and differentiation.

PCa, the most common male cancer in the developed world is a leading cause of mortality^4^, but with a relatively unknown aetiology with age, diet and ethnicity being strong risk factors. Prostate carcinogenesis is linked to chronic inflammation often caused by high dietary fats and heterocyclic amines or unknown pathogenic infections^5^. By the time PCa becomes symptomatic, hormonal therapy or invasive surgery may be the only avenues available, indicating a clinical need for early disease detection and to identify genetic changes earlier.

Long term exposure to chronic inflammation has been linked to cancer, and miRs are strongly associated with the inflammatory response. Several miRs are regarded as being immune cell modulators or induced by immune cell responses, e.g., miR-155 is induced in the macrophage^6^ and miR146 in monocytes^7^ respective inflammatory responses, and they can modulate both the innate and adaptive immune systems^8,9^. Several authors have put forward models which involve oncomiRs secreted by cancerous tissues (via exosomal transfer or Aurgonaute2-bound) being detected by the immune cell TLR-8 (Toll like receptor) which trigger an inflammatory response involving IL-6 and TNFα, which can accelerate tumour growth^10,11^. However, high levels of miR expression in tumour tissue have been attributed as immune cell derived—as a consequence of inflammatory signalling, and it is unclear how much miR cross-talk occurs between immune and tumour cells. Therefore, identifying the specific cellular source of the miRs is also of paramount importance, as is their downstream effects on the expressed mRNA transcriptome.

In cancers such as PCa, with unknown aetiology, knowing the primary source of the miR signalling may be important. Oncogenic pathways can activate several transcription factors—each able to upregulate a cohort of microRNAs, each able to drive forward carcinogenesis. However, immune cells responding to chronic inflammation within a non-transformed tissue may eventually promote carcinogenesis with the concurrent/co-incidental activation of miR responses.

Profiling experiments using human PCa samples show a high degree of miR heterogeneity—due to variance in sample collection, processing and the oncogenes/tumour suppressors driving the disease^12–14^, as well as potentially confounding clinical issues. Additionally, samples of human early PCa or prostate intraepithelial neoplasia are difficult to obtain due to few surgical resections at this asymptomatic stage.

High-grade prostatic intraepithelial neoplasia (PIN) is accepted as a plausible precursor of PCa based on clinical and histologic features; both share the same molecular alterations and develop from the androgen receptor (AR) positive luminal epithelial cell layer and can include TMPRSS2^15^ fusions and *PTEN* deletions^16^. *PTEN* is one of the most frequently mutated/deleted genes in human cancers, found in 30%+ of all human primary PCas, and 60%+ of higher grade metastatic lesions^17–19^. Evidently, PTEN may have multiple roles both in tumour initiation and tumour progression.

The PI3/AKT pathway controls important pathways in cell cycle regulation, proliferation, and cancer, and activates proteins and transcription factors which function in proliferative pathways along with phosphoinositides (PIP_3_) with second messenger functions^20,21^. PTEN (phosphatase and tensin homologue deleted on chromosome 10) functions to antagonise the PI3/AKT pathway. PTEN is a phosphatase which catalyses the dephosphorylation of the 3′ phosphate of the phosphatidylinositol (3,4,5)-trisphosphate (PIP3), resulting in the inhibition of the AKT signalling pathway^22,23^. In late stage PCa, when hormonal therapeutic avenues become ineffectual, the PI3/AKT pathway (activated by *PTEN* loss) remains an active therapeutic intervention avenue, targeting one of the downstream PI3/AKT substrates namely the mammalian target of rapamycin (mTOR).

Wang et al. established *Pten* ^loxp/loxp^ *:Pb*-*Cre*4 mice, which have conditional *Pten* alleles deleted by a Probasin (*Pb*) promoter-driven *Cre* recombinase, limiting *Pten* deletion to the prostatic epithelial layer^24^. *Pten* deletion leads to enlarged prostate glands with an accelerated development of mouse PIN by 10 weeks, with invasive adenocarcinoma thereafter. Although the mouse prostate structure is different to human, there are several points of commonality—specifically with the glandular epithelial AR-driven structure.

Here we have utilised this *Pten* knock out mouse model, and epithelial cells derived from it, to study the source and production of specific miRs in the development of PIN and to assess whether certain miRs may signal the onset of early PCa, and how they may promote the disease.

## Results

### Characterisation of mouse prostate tissues

We utilised the *Pten* ^*-/-*^ model for our primary studies as factors, e.g., age, diet, stage, genetics, tissue collection and processing could be kept tightly controlled. Additionally, mice would be kept in a sterile environment with no mating partners—minimising or ruling out any acquired pathogenic sources of prostatitis.

To investigate miR expression in *Pten* deletion-induced prostate tissue at PIN, we collected prostate tissues from *Pten* ^loxp/loxp^;*PB* ^Cre4+ve^—(henceforth called *Pten* ^−/−^ or *Pten* ^ko^) mice and compared them to prostate tissues of age matched *Pten* ^loxp/loxp^;*PB* ^Cre4-ve^ essentially wild type mice. 8 *Pten* ko and 11 *Pten* wild type mouse prostate tissues were obtained.

**Fig. 1:**
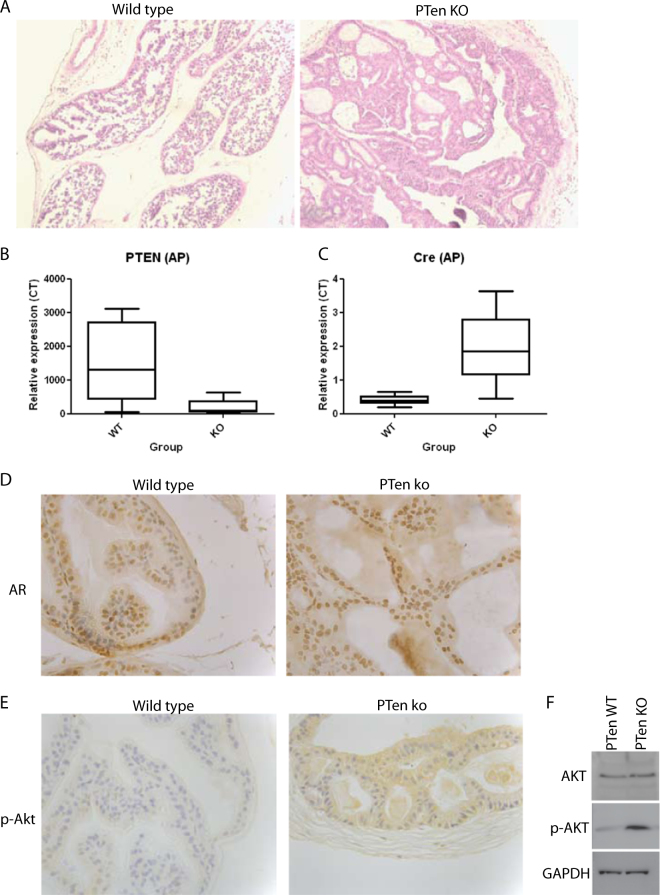
Characterisation of the ***Pten*** deletion mouse prostate tissue **a** Hematoxylin and eosin staining of formaldehyde fixed wax-embedded prostate tissues taken from the *Pten* ^*loxp*/loxp^; *Pb-Cre*4^+ve^ (*Pten* KO) and *Pten* ^*loxp*/loxp^ (*Pb-Cre*4^-ve^) 'wild type' mice. **b** Boxplot showing qPCR analysis results of *Pten* mRNA levels (at exon 5) from total RNA obtained from *Pten* ^−/−^ and wild type mouse prostate tissues (results shown for the anterior prostate = AP). **c** Boxplot showing qPCR analysis results of *Cre* expression levels from total RNA obtained from *Pten* ^−/−^ and wild type mouse prostate tissues. Results represent the means of *n* = 11 tissues measured in triplicate. **d** Immunohistochemical staining for the prostate epithelial marker—androgen receptor (AR) in *Pten* ^−/−^ and wild type mouse prostates. **e** Immunohistochemical staining for phospho-AKT in *Pten* ^ko^ and *Pten* ^wt^ tissue sections. **f** Western blot of phospho-AKT and total AKT from protein extracts of mouse prostate tissues

*Pten* ^−/−^ prostate tissue examination revealed hyperplasia and characteristics associated with PIN (see Fig. 1a). No invasion outside the prostate capsule was observed. The *Pten* deletion was validated by quantitative polymerase chain reaction (qPCR; see Fig. 1b, c). The prostate epithelium-specific dysregulation was also evaluated by immunostaining with the prostate epithelial marker—AR, see Fig. 1d. Phospho-Akt expression was also elevated in the *Pten* ^−/−^ tissues, see Fig. 1e, f, indicative of an overactive Akt pathway.

### Identification of microRNAs whose expression is changed in ***Pten***^−/−^ mouse prostate tissue

We examined the miR expression (750+ miRs) from a cohort of prostate tissues from both groups. Four age-matched pairs of mouse tissue were used for global microRNA expression analysis. Samples did not show a high degree of variation in global sample Ct values indicating sample reproducibility.

**Fig. 2:**
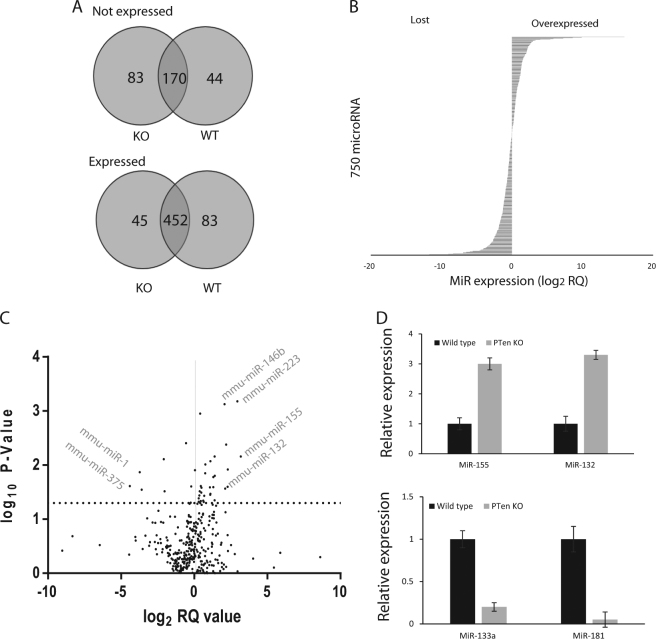
MicroRNA expression levels are significantly changed in ***Pten*** deletion-driven prostate intraepithelial neoplasia (PIN) **a** Venn diagram indicating expressed and non-expressed miRs in the *Pten* ^−/−^ (KO) and wild type (WT) prostate tissues. Data obtained from the Taqman low density array *n* = 4. **b** Bar chart indicating the expression levels of all 750 miRs in the *Pten* ^−/−^ prostate tissues—expressed as log_2_ relative quantification (RQ) over wild type. **c** Volcano plot indicating the expression profile and *p* values of all 750 miRs from the LDA card analysis (dotted line indicates *p* value at 0.05). **d** Independent validation of the expression levels of a sample of miRs by qPCR, in the main complete cohort. Mir expression was normalised to sno-RNA-234 & 202. Data represent the means the replicates from *n* = 8 wt and 11 ko tissue samples

From the 750 microRNAs studied, 452 were detectable and 170 were undetectable in both groups (see Fig. 2a). Forty five microRNAs showed *Pten* ^−/−^-specific expression, and 83 showed a complete loss of expression in the *Pten* ^−/−^ prostates (see Fig. 2a). Figure 2b shows the expression characteristics of all 750 miRs compared to wild type prostate tissue. Figure 2c shows a volcano plot of all microRNAs along with their expression levels and *p* values. From the 452 miRs expressed in both groups, 39 miRs showed expression with strong p values <0.05 (see Fig. 2c and Table 1). Sixteen miRS were found to be significantly upregulated in *Pten* ^−/−^ prostates (2-fold or higher) and five were found to be significantly downregulated (−2-fold or lower). A list of the top 20 miRs lost or expressed in *Pten* ^*-/-*^, uniquely, are given in Supplementary Table A, although no *p* values are given due to the lack of expression in the other group. The microRNAs mmu-miR-155 and mmu-miR-132 were validated by qPCR of the whole cohort of mouse tissues (ko = 8, wt = 11) (see Fig. 2d, upper panel) and were found to be overexpressed by over 3-fold in *Pten* ^−/−^ prostates. Similarly mmu-miR-133a and 181 were found to be reduced (see Fig. 2d, lower panel).

**Table 1:** MicroRNA expression values (relative quantification) from ***PTen*** ^−/−^ mouse prostates compared with wild type

MicroRNA name	RQ value	*p* value
mmu-miR-155	9.11	0.007
mmu-miR-223	7.74	0.001
mmu-miR-146a	4.91	0.012
mmu-miR-132	4.87	0.025
mmu-miR-142-3p	4.53	0.004
mmu-miR-135b	4.33	0.027
mmu-miR-146b	4.20	0.001
mmu-miR-150	2.64	0.016
mmu-miR-34b-3p	2.64	0.007
mmu-miR-31	2.63	0.045
mmu-miR-16	2.47	0.026
mmu-miR-126-3p	2.47	0.017
mmu-miR-210	2.16	0.034
rno-miR-532-5p	2.14	0.008
mmu-miR-342-3p	2.13	0.035
mmu-miR-409-3p	2.11	0.026
mmu-miR-139-5p	1.70	0.010
mmu-miR-339-3p	1.68	0.014
mmu-miR-134	1.60	0.037
mmu-miR-21	1.57	0.048
mmu-miR-411	1.53	0.045
mmu-miR-429	1.41	0.026
mmu-miR-127	1.40	0.032
mmu-miR-191	1.37	0.046
mmu-miR-188-5p	1.37	0.024
mmu-miR-186	1.33	0.001
mmu-miR-434-3p	1.30	0.031
mmu-miR-199a-3p	1.27	0.036
mmu-miR-431	1.24	0.041
mmu-miR-532-3p	1.23	0.048
mmu-miR-126-5p	1.03	0.012
mmu-miR-130a	0.87	0.025
mmu-miR-376c	0.85	0.050
mmu-miR-324-5p	0.68	0.004
mmu-miR-384-5p	0.25	0.030
mmu-miR-221	0.24	0.008
rno-miR-1	0.08	0.028
mmu-miR-1	0.08	0.014
mmu-miR-375	0.05	0.024

List includes all microRNAs with *p* values of >0.05

### Identification of genes whose expression is changed in ***Pten***^−/−^ mouse prostate tissue

**Fig. 3:**
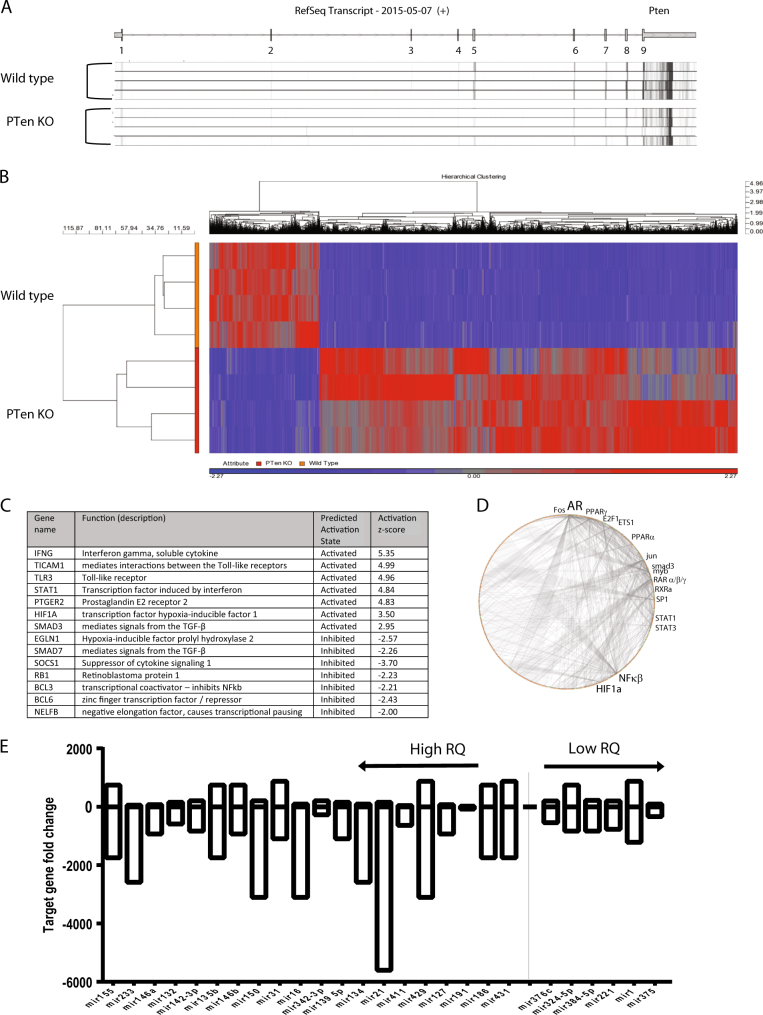
***Pten*** deletion significantly alters the expression of over 4000 genes in ***Pten***^−/−^ prostate intraepithelial neoplasia (PIN) **a** Heat map of mRNA transcript fragment reads from *Pten* ^*loxp*/loxp^; *Pb-Cre*4^+ve^ (*Pten* KO) and *Pten* ^*loxp*/loxp^ (*Pb-Cre*4^-ve^) 'wild type' prostate-derived mRNA (*N* = 4 replicates) overlayed on the gene structure of mouse *Pten* (RefSeq, 2015)—indicating the absence and/or reduction of *Pten* RNA fragments at exon 5. **b** Diagram showing the hierarchical clustering analysis of the 4000+ genes either upregulated or downregulated (±2-fold, *p* = <0.05) showing the four replicates (drawn in Partek). **c** Table indicating the most activated and inhibited upstream regulators likely to be responsible for the genetic changes observed. Data presented from Ingenuity Upstream Regulator analysis. **d** Diagram indicating the shared regulatory interactions of the transcription factors involved in the genetic changes observed in the *Pten* ^−/−^ prostates (drawn using http://molbiol-tools.ca/Transcriptional_factors.htm). Font size indicates increasing interactions. **e** Bar chart showing the average fold change (expression) of the predicted mRNA targets of the miRs found overexpressed (High RQ) or lost (Low RQ) in the *Pten* ^−/−^ RNAseq cohort. Graph is ranked according to RQ of the expressed miR of interest (*p* < 0.05). Target predictions done in with www.microRNA.org and Partek (with Targetscan) software. Mir targets *n* = 200–600 genes approx

To analyse the effect of the *Pten* ^*-/-*^-induced changes in miR expression on the mRNA transcriptome, we carried out RNA-seq on the same RNA samples as analysed in Fig. 2 (*N* = 4 in each group). Figure 3a shows a map of the RNA-fragment aligned reads overlaid on the gene structure of mouse *Pten* and together with Fig. 1c confirms the strong reduction or absence of reads at *Pten* exon 5 (the remainder of exon 5 reads presumably coming from remaining contaminating stromal, endothelial, immune and blood cells etc not expressing *Pb-Cre*). Hierarchical cluster analysis on the four replicates showed a good separation between sample groups (see Fig. 3b). We observed that at PIN, the *Pten* deletion resulted in the change of over 4000 genes (*p* > 0.05, 2-fold change) as summarised in the heat map with hierarchical clustering. 3321 genes showed more than +2-fold increase in expression, while 831 genes showed a −2-fold reduction in expression in *Pten* ^−/−^ prostates. The top 20 up and down regulated genes are given in Supplementary Tables B–E. Supplemental Fig. 1a shows q-PCR validation of the expression of a subset of these genes in the original cohort set (ko = 8, wt = 11). When compared to the data presented by Wang et al., overall there was a strong agreement in the gene expression profiles for both studies (*R* = 0.76), with the directionality of gene expression being very similar (see Supplemental Fig. 1b).

### Functional categories and pathways for genes altered by *Pten* deletion in the prostate

Functional gene ontology analysis (KEGG) of the 4000 genes changed in the *Pten* ^−/−^ prostate tissue clustered very strongly into key pathways, mainly centred upon immunological responses, e.g., host tissue hyper-proliferation, inflammatory signalling and infection responses (see Fig. 3c). These gene pathways were driven by Toll-like receptors, interferon-γ (IFNG) and prostaglandin receptor (PTGER2).

Upstream regulator analysis (Igenuity Knowledge Base) revealed several key transcription factor candidates likely for the gene expression pattern observed including AR, NF-κΒ, HIF1α, Stat1&3, Fos, Jun, YY1 and Myb. Additionally, the nuclear receptors PPAR-γ/α and RAR-α/β/γ and RXRα had significant inputs (see Fig. 3d).

The gene set observed in the RNAseq analysis (*p* > 0.05, -/+2), was then analysed for micro-RNA binding sites. The average gene expression levels were plotted against each significantly regulated microRNA target. Gene expression data showed that mRNAs with target regions in their UTRs for the strongly overexpressed microRNAs, e.g., mir-155, 223 showed an average reduction in gene expression within *Pten* ^−/−^ prostates. Genes with binding sites for low or under-expressed miRs (low RQ) showed less downregulation (see Fig. 3e).

### The source of microRNA overexpression within the ***Pten***^−/−^ prostate originates from the inflammatory prostate epithelia and from infiltrating immune cells

We analysed the tissues by immunohistochemical staining for the upstream regulatory markers indicated by the Ingenuity pathways. This would allow us to determine the origin and drivers of miR overexpression in the tissue, i.e., *Pten* ^−/−^ driven or immune cell derived. Gene ontology and upstream factor analysis showed that the transcription factors AR, STAT3, NFκβ, HIF1α, and RAR/RXR as well as immunological mediators, e.g., interferon-γ, toll-like receptors (TLR7-8) and prostaglandin receptors were strongly implicated in the gene expression pattern observed.

**Fig. 4:**
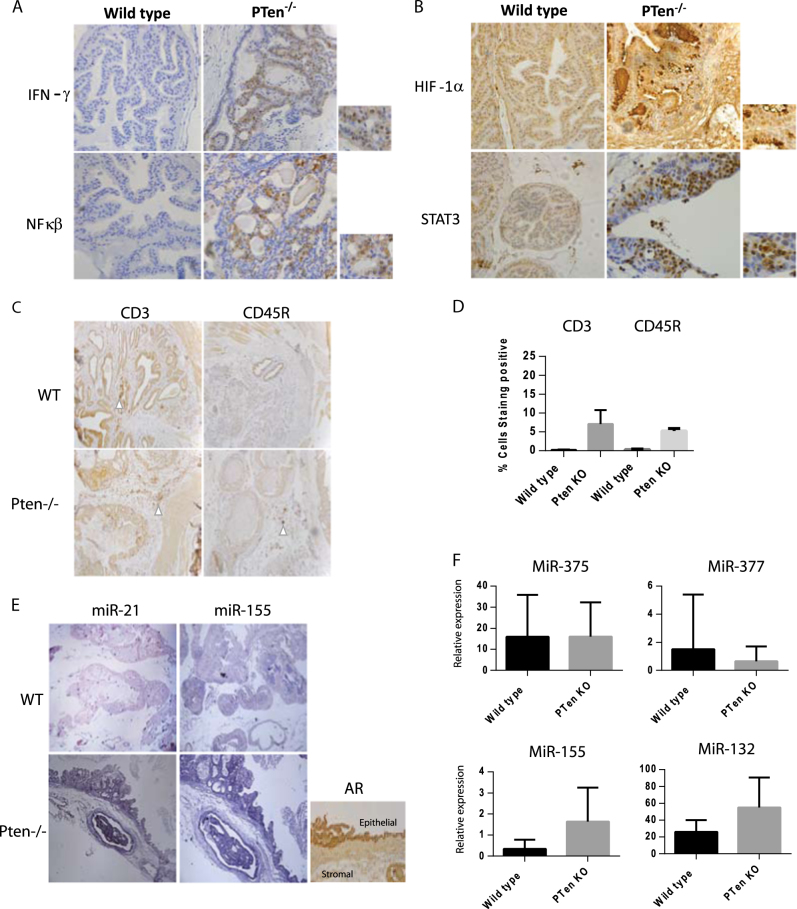
Inflammatory pathways are activated in ***Pten***^−/−^ prostate epithelial cells at the prostate intraepithelial neoplasia (PIN) stage **a** Immunohistochemical staining for IFN-γ and NF-κβ in *Pten* ^−/−^ (KO) and wild type prostate tissue. **b** Immunohistochemical staining for HIF1-α and STAT3 in *Pten* ^−/−^ (KO) and wild type prostate tissue. Magnified sections from *Pten* ^−/−^ shown inset on the right hand side. **c** Immunohistochemical staining for CD3 and CD45R in mouse prostate tissue. **d** Bar graph representing the % of cells staining positive for the immune cell markers CD3 and CD45R. Results indicate the number of staining cells in five random fields of view across the tissue section, compared to the total number of cells. **e** In situ hybridisation staining for the microRNAs mmu-miR-21 and mmu-miR-155 in *Pten* ^−/−^ (KO) and wild type prostate tissue. Right hand side inset panel indicates areas of epithelial AR staining for reference. **f** q-PCR analysis of expression levels of mmu-miR- 155, 132, 375 and 377 from the serum of *Pten* ^*loxp*/loxp^; Pb-Cre4^+ve^ (*Pten* KO) and *Pten* ^*loxp*/loxp^ (Pb-Cre4^-ve^) 'wild type' mice. Serum miR levels were normalised to a *C. elegans* miR-39 spike in control

Immunohistochemical analysis of wild type and *Pten* ^−/−^ prostate sections showed specific staining for IFN-γ, NF-κβ and STAT3 within the glandular epithelium of the *Pten* ^−/−^ tissues. (see Fig. 4a, b). HIF1α levels were slightly higher in *Pten* ^−/−^ tissues (Fig. 4b) and AR levels were equally strong in both tissue types (see Fig. 1d).

When analysing the immune cell infiltration within these tumour sections we observed that both CD3^+ve^ T cells and CD45R^+ve^ B cells were increased within the stromal compartment of the *Pten* ^−/−^ prostate tissue (10%+ of cells). Additionally, we observed an increase number of monocytes and macrophages via haematoxylin and eosin staining (see Supplemental Fig. 2). However, the immune cell infiltration did not correlate with the staining pattern seen for the inflammatory markers IFNγ, NFκβ, STAT3 or the epithelial marker AR. A low level of immune cell infiltration was seen within the hyperplastic epithelial cell layers (1% of cells approx.) (see Fig. 4c, d), whereas immune cells were located in stromal clusters, near blood vessels or were peripheral. Upon in situ hybridisation with anti-miR-155 and mir-21 probes, staining for miR expression was seen most strongly in the epithelial layer (see Fig. 4e), and not in the stromal, endothelial or immune cell infiltrates, indicating the source of the overexpressed miRs to be from the epithelial cells (as highlighted in the AR stained adjacent tissue section for epithelial cell reference). In wild type prostate sections—individual immune cells could be seen staining positive for miR-155/21 (0.5–1% immune cell infiltrate) within the normal unstaining prostate epithelium (see Fig. 4e). Interestingly, miR levels in the serum of the mice harbouring the *Pten* ^−/−^ prostate tissue showed elevated levels of the overexpressed miRs, although the exact cellular source of the serum miR cannot be specifically identified (see Fig. 4f).

### ***Pten*** deletion drives miR overexpression from the epithelium in vitro

To investigate whether the overexpressed miRs observed in the *Pten* ^−/−^ tissue were driven by *Pten* deletion or whether due to responding localised inflammatory immune cell involvement, we studied the *Pten* ^−/−^ prostate epithelial cells in isolation, in culture.

**Fig. 5:**
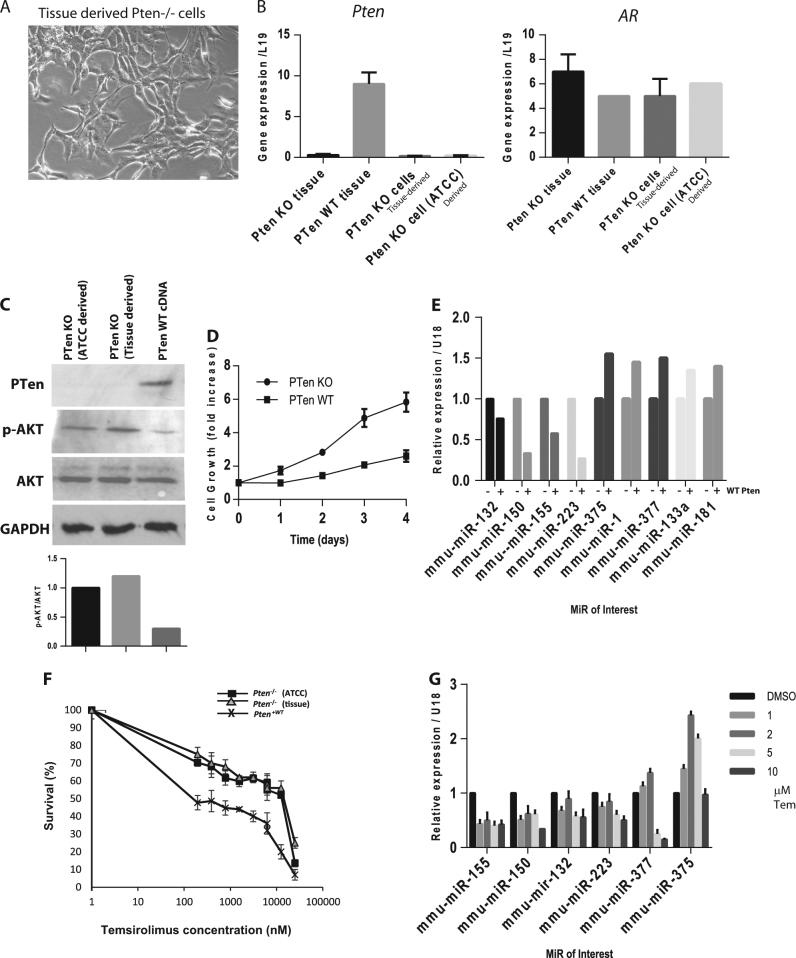
MiR overexpression is maintained in ***Pten***^−/−^ prostate epithelial cells in vitro and is inhibited by wild type ***Pten*** re-introduction and mTOR inhibitor—temsirolimus **a** Phase contrast microscope image of isolated *Pten* ^−/−^ cells from *Pten* ^*loxp*/loxp^; Pb-Cre4^+ve^ (*Pten* KO) mouse prostates. **b** qPCR expression levels of *Pten* (left hand side) and *AR* (right hand side) in the tissue derived from adult male *Pten* ^*loxp*/loxp^; Pb-Cre4^+ve^ (*Pten* KO) or wild type mice, compared to the *Pten* ^−/−^ mouse tissue-derived prostate cells or cells derived from the ATCC directly (*Pten* ^*loxp*/loxp^;*Cre* ^+ve^). **c** Western blot analysis of PTEN, AKT and phospho-AKT levels in *Pten* ^−/−^ cells stably transfected with wild type *Pten* (pEF6-*Pten* ^*wt*^ expressing plasmid). Densitometry data for p-AKT/AKT is given in the bar graph underneath. **d** Crystal violet cell growth assay of the *Pten* ^−/−^ and *Pten* ^+wt^ cell lines over time (4 days). Error bars represent the means of three independent replicates. **e** qPCR analysis of a panel of miR expression in *Pten* ^−/−^ and *Pten* ^wt^ cell lines. Results represent fold change from *Pten* ^−/−^. Error bars represent the standard error from three independent replicates. Expression normalised to mouse small RNAs sno-202 and sno-234. **f** Crystal violet cell growth assay of *PTen* ^−/−^ cells (either tissue derived or ATCC) treated with increasing doses of Temsirolimus for 96 h, compared to *Pten* ^+WT^ cells. Data are plotted as % survival compared to DMSO control. **g** qPCR analysis of a cohort of miRs from *PTen* ^−/−^ cells treated with the mTORC1 inhibitor Temsirolimus (1–10 μM versus DMSO vehicle) for 16 h. Results represent fold change in expression over DMSO vehicle

We cultured isolated prostate epithelial cells from the *Pten* ^−/−^ mice which expanded into a cell line rapidly within 4–5 passages (see Fig. 5a). The *Pten* and *AR* levels were measured by qPCR, as well as compared to wild type prostate tissue and mouse prostate *Pten* ^−/−^ cells acquired from the ATCC cell bank (see Fig. 5b). The tissue-derived *Pten* ^−/−^ cells were indistinguishable from the ATCC-PTEN-CAP8 cell line by these criteria. No CD3 or CD45R expression could be detected on western blot extracts (data not shown). MiR expression was high in these cell lines when compared to wild type tissue (see Supplemental Fig. 3).

To evaluate the effect of *Pten* on miR expression in vitro, we than stably transfected the *Pten* ^−/−^ cells with a pEF6-*Pten* ^*WT*^ vector, to re-introduce wild type *Pten* expression. An increase in full-length PTEN protein was observed, and cells also showed the inhibition of the phospho-AKT pathway in these wild type *Pten* expression cells (see Fig. 5c). These cells also showed a reduced cell growth (see Fig. 5d).

The re-introduction of wild type *Pten*, strongly reduced the expression of the overexpressed miRs 132, 150, 155, 223 but increased the expression of the previously downregulated miRs-375, mir-377 and miR-1 (see Fig. 5e). Wild type *Pten* also increased the sensitivity of these cells to the rapamycin analogue Temsirolimus (Fig. 5f). The mTOR protein regulates several downstream transcription factors which could be regulating the microRNA transcription, and mTOR inhibitors can reverse many aspects of the *Pten*-transformed phenotype^25^. Temsirolimus treatment resulted in the strong reduction of miR-155, 132, 150 and 223 levels by 30–50%, and interestingly, miRs-377 and 375, found to be downregulated in *Pten* ^−/−^ prostate tissue, showed a biphasic response first increasing in expression then reducing (see Fig. 5g).

**Fig. 6:**
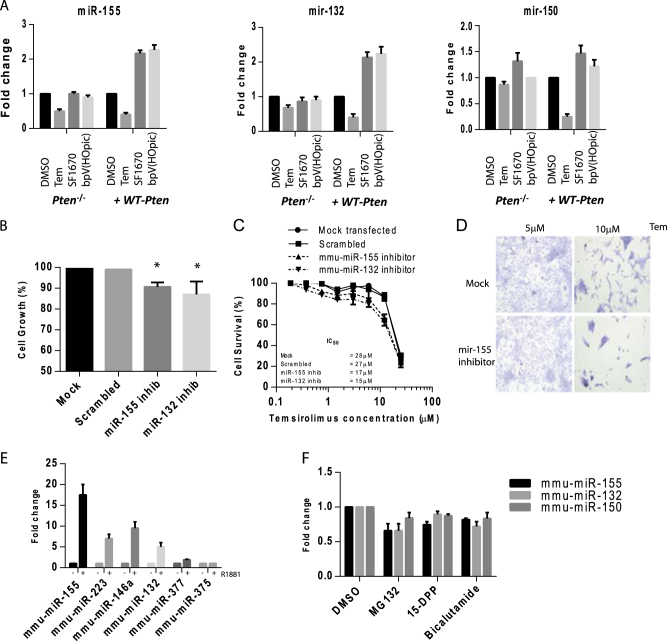
Re-introduction of wild type ***Pten*** into ***Pten***^−/−^ prostate cells and inhibition of the PI3kinase pathway reduces miR overexpression in vitro **a** qPCR analysis of miR-155, 150 and 132 expression levels in both *Pten* ^wt^ and *Pten* ^−/−^ cells in response to treatment with mTORC1 inhibtor Temsirolimus and PTEN inhibitors SF1670 and bpV(HOpic). **b** Crystal violet cell growth assay of *Pten* ^−/−^ tissue-derived cells transfected with a mir-155 or mir-132 inhibitor (or scrambled control) for 48 h. Data are plotted as % cell survival normalised to mock transfected cells. **c** Crystal violet cell growth assay of *Pten* ^−/−^ cells transfected with a mir-155 or mir-132 inhibitor (or scrambled control) and treated with increasing doses of temsirolimus. Data represent the % survival as compared to DMSO after 96 h. **d** Microscopy photograph of formalin fixed, crystal violet stained, *Pten* ^−/−^ cells transfected with miR-155 inhibitor oligo and treated with 5 or 10 μM Temsirolimus for 96 h. **e** qPCR analysis of microRNA expression in *PTen* ^−/−^ cells hormonally starved for 72 h and then treated with R1881 (10 nM) for 8 h. Data are normalised to mouse small RNAs sno-202 and 234. **f** qPCR analysis of microRNA expression in *PTen* ^−/−^ cells treated with MG132 (NFκβ inhibitor), 15-DPP (STAT3 inhibitor) and bicalutamide (AR antagonist) for 8 h. Data are normalised to mouse small RNAs sno-202 and 234. *p value 0.05

Analysing miR expression in *PTen* ^wt^ prostate cells treated with PI3K/PTEN modifying drugs showed that the mTORC1 inhibitor (Tem) decreased miR-155, miR-150 and miR-132 expression, while inhibition of the ectopically expressed wild type PTEN with the highly selective PTen inhibitors SF1670 [N-(9,10-Dihydro-9,10-dioxo-2-phenanthrenyl)-2,2-dimethyl-propanamide] and bpV(HOpic) [Dipotassium bisperoxo (5-hydroxypyridine-2-carboxyl) oxovanadate] increased miR expression (Fig. 6a).

When *PTen* ^−/−^ cells were treated with miR-155 and 132 inhibitors, we saw a modest but statistical significant reduction in cell growth over 4 days (−8%), compared to mock or scrambled controls (see Fig. 6b). When combined with Temsirolimus treatment we observed a synergistic effect, where mir-155 or 132 inhibitor treatment resulted in decreased cell growth and an IC_50_ reduction of Temsirolimus from 28 to 15 μM approx (see Fig. 5c, d).

As well as mTOR, we also investigated other potential drivers of miR expression, e.g., AR, NFκβ and STAT3. The androgen induction of these microRNAs was evaluated by treating hormonally starved *PTen* ^−/−^ cells (72 h) with the synthetic androgen R1881. qPCR analysis of microRNA levels showed that androgen-stimulated cells strongly increased their mir-155, 223, 146a, 132 expression while mir-375 and 377 were unaffected (see Fig. 6e). The anti-androgen bicalutamide reduced this expression (see Fig. 6f). The NFκβ inhibitor (non-specific) MG132 reduced mir-155, 132, and 150 expression, as did the STAT3 inhibitor 15-DPP (see Fig. 6f).

**Fig. 7:**
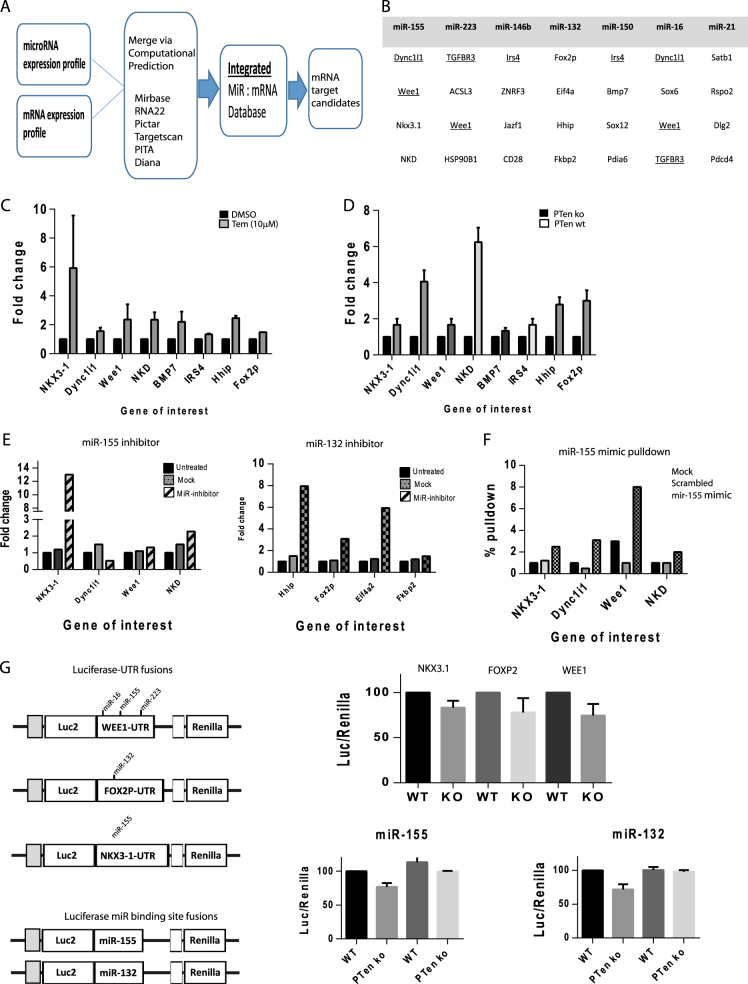
Identification of mRNAs targeted by the inflammatory miRNAs in ***Pten***^−/−^ cells **a** Schematic diagram of the computational pathways utilised to predict miR:mRNA interactions/targets. **b** Table of the five most likely downregulated mRNA targets predicted by over 4/6 miR databases. **c** qPCR analysis of a cohort of genes from *Pten* ^−/−^ tissue-derived cells treated with 10 μM temsirolimus for 24 h. Gene expression is normalised to *Rpl*19, *Gapdh* and *β-actin*, and then plotted as fold change over DMSO treatment. **d** qPCR analysis of a cohort of genes from either *Pten* ^−/−^ or *Pten* ^+WT^ cells. Gene expression is normalised to RPL19, GAPDH and β-actin, and then plotted as fold change over *Pten* ^−/−^. **e** qPCR analysis of a cohort of genes from *Pten* ^−/−^ tissue-derived cells transfected with miR inhibitor oligos (or mock transfected or transfected with scrambled oligo). Left hand side = miR155 inhibitor, right hand side = miR132 inhibitor. Gene expression is plotted as fold increase/decrease over untreated cells (normalised to *Rpl*19, *β-actin* and *Gapdh*). **f** qPCR of a cohort of genes from RNA purified from *Pten* ^−/−^ cells transfected with a mmu-mir-155 biotinylated mimic and RNA pulldown assay using streptavidin beads. Cells were transfected with biotinylated scrambled control oligo. Data are normalised as % pulldown compared to mock transfected cells. **g** Left hand side—schematic diagram of the luciferase-3′-UTR plasmid constructs and 3′miR binding site luciferase fusions. Right hand side—Dual luciferase assays from extracts of *Pten* ^−/−^ (KO) and *Pten* wild type (WT) mouse prostate cells transfected with the luciferase UTR constructs or miR binding site constructs for 48 h. For miR fusion reporters Pten KO or WT cells were also treated with 10 μM temsirolimus. Luciferase activity is normalised to the constitutively active renilla luciferase

We then validated if the downregulated genes seen in the *PTen* ^−/−^ prostate cells were in fact regulated or influenced by the aberrant *Pten* ^−/−^ pathway-induced miR upregulation. In silico analysis using the miR:mRNA expression profiles using six online databases (see Fig. 7a) indicated a potential list of candidate genes—several of which were targeted by multiple miRs, e.g., *Wee1* (see Fig. 7b).

Firstly we treated *PTen* ^−/−^ cells with temsirolimus, which strongly inhibited mir-155, 132, 150 and 223 expression (see Fig. 5g). qPCR gene expression analysis showed an increased expression of a panel of eight genes of interest (see Fig. 7c). Similarly, the *PTen* ^wt^ expressing cell line showed very similar increases in gene expression (see Fig. 7d). We transfected *Pten* ^−/−^ cells with LNA-modified miR-155 or 132 inhibitors and analysed expression of our predicted target genes. MiR-155 inhibitor treatment increased the expression of *Nkx3-1*, *Nkd*, *and Wee1*, while the miR-132 inhibitor increased the expression of *Hhip*, *Fox2p*, and *Eif4a2*. The proposed miR target genes *Dync1i1* and *Fkbp2* showed no significant changes (see Fig. 7e). Mir-155 biotinylated pulldown analysis of mRNA transcripts bound to miR-155 showed an enrichment of *Nkx*3-1, *Dync1i1*, *Wee1* and *Nkd* mRNA (see Fig. 7f).

Finally we constructed firefly luciferase reporters fused with either full length 3′-UTR regions of three genes of interest, namely *Wee1*, *NKX*3.1 and *Fox2p* or fusions containing the pure miR binding site for miRs 155 and 132 (see Fig. 7g). When transfected into mouse prostate cells we measured a lower activity of the luciferase reporter as compared to the constitutive renilla reporter, expressed from the same plasmid, of 10–20% approx. Further, the reduced luciferase activity of the pure miR binding site reporters were abrogated when miR overexpression was inhibited by temsirolimus treatment.

When we transfected expression plasmids (pEF6) expressing *Wee1*, *Nkx*3.1 or *Fox2p* into mouse *PTen* ^−/−^ cells we observed a modest reduction in cell growth over 6 days—indicating the normally repressive function of these genes (see Supplemental Fig. 4).

## Discussion

*PTen* deletion produces an overactive PI3 kinase pathway driving forward proliferation, thus its targeting is an attractive treatment avenue for late stage PCa that become hormone refractory. Several clinical trials have evaluated such agents in these PCa patients with only modest success. However, patient stratification for *PTen* status is not currently routine.

Although structurally different, mouse and human prostates are histological similar as are the drivers of prostate gland development. Prostate epithelial cell-specific *PTen* deletion in the mouse leads to the development of hyperplasia and PIN, leading to adenocarcinoma and advanced disease. The abnormal prostate growth is initially hormone sensitive, which also become refractory with time—recapitulating several aspects of the human disease.

### Downstream effects of ***PTen***^−/−^ on gene expression

We examined the effect of *PTen* loss in the mouse prostate on expression of mRNA and miRNAs. Even at the PIN stage, we observed a significant change in over 4000 genes and up to 40 miRs.

The upregulated microRNAs found here have been extensively identified in disease and cancer-related pathways. However, several of our overexpressed miRs including miRs-155, 146a/b, 132, 223, 21 and 16 have also been identified as being Toll-like receptor-inducible (reviewed in ref. ^26^), and several identified as transcribed by NF-κΒ and Fos/Jun^27^. MiR-155, our highest overexpressed miR, is conserved across mammals^28^. It is prominently expressed across haematopoietic cells and identified as an oncomiR in haematological malignancies, breast, lung and colon cancer^29^. MiR-155 is a consistent feature of mammalian inflammatory responses^6^, often initiated by Toll-like receptor pathways. However, miR-155 has been investigated in PCa, and found to be upregulated, with roles targeting *TGFβ/SMAD*, *Annexin*7 and the mismatch repair genes *hMLH1/hMSH6* ^30–32^ in conjunction with miR-21—also upregulated here. Other targets include genes involved in apoptotic resistance and the homeobox-containing transcription factor *NKX3*-1—discussed below. MiR-155 has also been implicated in reprogramming normal tumour-adjacent fibroblasts to cancer associated fibroblasts via exosome signalling in both prostate and pancreatic cancers^33,34^—relevant as miR-155 was increased in the sera of *Pten* ^−/−^ mice.

Other overexpressed miRs found in our study have also been strongly implicated in PCa. MiR-132 expression is associated with inflammation, inducing proliferation of endothelial cells in the tumour environment^35^. Mir-223 has been found to be upregulated in PCa tissues^36^, and the oncomiR miR-21, is associated with poor biochemical recurrence-free survival in PCa patients^37^. High mir-150 expression is positively correlated with tumour recurrence and metastasis^38^.

The downregulated or lost miRs in our study, such as miR-1 and its cluster partner miR-133, are tumour suppressor miRs, previously identified as consistently downregulated in primary prostate tumours^39^. MiR-375, also identified as a tumour suppressor^40^ showed a strong downregulation our study.

Infiltrating immune cells did show some mir155 staining but their numbers and locations did not correlate with the staining pattern observed. We observed that these miRs were produced very early in prostate hyperplasia, and are derived from the epithelial cells themselves. They are secreted, and may influence surrounding cell growth via inflammatory interactions with the immune system and trigger oncogenic pathways in the complex primary tissue environment. Although there is a strong crossover with inflammatory responses for several of the overexpressed miRs in our study, these have also been identified as overexpressed in cells growing in vitro by ourselves and other groups. OncomiR expression is retained when cells are removed from their primary tissue of origin, and here we have shown that they are primarily driven by events such as *PTen* loss, and not as a result of immune cell signalling. Human prostate cells growing in culture showed highest miR-155 levels in the more aggressive cell lines, e.g., PC3 (see Supplemental Fig. 5).

### Gene analysis and pathway enrichment

Pathway enrichment analysis of genes in *PTen* ^−/−^ tissue showed clustering upon carcinogenesis and immunomodulatory pathways. Upstream analysis of both transcription factors and other regulatory molecules indicated a strong involvement of AR, STAT1&3, NF-κΒ and HIF1α as being responsible for the gene expression pattern. PTEN regulates and increases the activity of these transcription factors.

PTEN activates HIF1α via the inactivation of Forkhead transcription factors^41^. HIF1α activation is an early event in prostate carcinogenesis, including mouse models^42–44^, and studies have also demonstrated that hypoxic responses are significantly increased in *PTen* null prostate cells—triggering cytokine response networks^45^.

STATs represent downstream effector transducers of the inflammatory cytokine and growth factor signalling pathways^46^ via mTOR and are implicated the promotion and progression of PCa^47,48^. STAT3 links inflammatory pathways to cancer development via miR-21, miR-155, miR-16 upregulation and NK-κΒ^49,50^.

AR is essential for prostate functional and developmental pathways. AR may be inactivated by an overactive AKT pathway^51,52^. Indeed, a strong downregulation in several AR-regulated genes, e.g., *Nkx*3-1, *Probasin*, and *Tgm*4 was seen, without AR protein levels changing. However, conversely the miRs studied here showed a strong upregulation by androgen-activated AR. AR has also been strongly correlated with upregulation of miR at the transcriptional level and during mature strand processing^53,54^—therefore the functioning of the AR remains conflicting in the PTen^−/−^ prostate.

Upstream regulatory analysis predicted that interferon-γ, Toll-like and prostaglandin receptors to be activated, resulting in the enrichment of immunological response pathways seen. PCa progression in humans has been strongly associated with chronic inflammation^55–57^, and Garcia et al.^58^ showed that loss of PTen in the mouse prostate epithelium leads to a significant upregulation of genes within the inflammatory and cytokine-to-cytokine signalling network. Other cancers with *PTen* deletions show similar patterns^59,60^.

miRs may regulate up to 90% of the genome^61^. Overall the miR:mRNA correlations showed on a broad level that the overexpressed miRs in *PTen* ^−/−^ showed a significant reduction in expression of their target genes, and a 'signature' of the overexpressed miRs could be seen in the regulated mRNA population.

Other gene downregulation changes in the *PTen* ^−/−^ prostate included—gasdermin family members, involved in inflammatory defence; *Bmp*7—involved in prostate morphological development; and *Kiss*1—involved in suppressing metastasis. Gene upregulation changes included—*Tff*3 (Trefoil factor 3) an epithelial secreted protein associated with mucosal defence, injury and carcinogenesis; *Wnt*4—essential for prostate development; *Fam*129b—a WNT family regulator; *Epst*11—associated with epithelial to mesenchymal transition in cancer and *Cxcl*16—chemokine strongly associated with inflammation in PCa.

### Inhibiting the PI3 kinase pathway

We tested whether the overexpressed miRs would (i), still be highly expressed in vitro and (ii), be responsive to PI3kinase pathway inhibitors. Additionally, we re-introduced wild type *PTen* cDNA into these cells and monitored their miR expression levels. The overexpressed miRs were still highly expressed in vitro, and these miRs could be reduced by Temsirolimus treatment, and by re-expressing wild type *PTen*. Conversely, the previously repressed miRs in *PTen* ^−/−^ cells, were increased by both treatments—indicating a direct role of the PI3 kinase pathway in controlling miR expression. The PTen inhibitor agents SF1670 and bpV(HOpic) increased the miRs’ expression in the PTen^wt^ cells. These treatments also increased the expression of a previously *PTen* ^−/−^—repressed gene cohort. Blocking the activity of the target miRs via miR-inhibitors also increased the expression of these target genes, although to a modest level.

Some of the miRs found to be overexpressed in this study were upregulated by the PI3 kinase pathway and are in part responsible for the reduction of certain target genes. An example being the miR-155 target gene—*Nkx*3-1, which was previously non-detectable. Although *Nkx*3-1 has been described as being silenced by methylation^62,63^, it is possible that miRs also play a role in the silencing of such genes—especially given that the luciferase-*Nkx*3.1 3′-UTR fusion reporter showed a reduction in expression. Indeed decreased NKX3-1 has been observed in areas of prostate inflammation in the aging prostate, and our data would support the role of inflammation-regulated miRs (e.g., miR-155) in this process^64,65^.

We tested a miR-inhibitor, individually and in combination with Temsirolimus—which reduces PTen-driven tumour subtypes in mice^66^. *PTen* is frequently mutated in castration-resistant PCa patients and in late stage metastasis^67^, and PI3k/mTOR inhibitors have been promised as chemotherapeutic agents, but with limited activity^68^. The miR-155 and 132 inhibitors showed a modest activity in the *PTen* ^−/−^ cells, and showed synergy with Temsirolimus, reducing the IC_50_ by almost 40%.

## Conclusions

Here we have observed that *PTen* deletion in the prostate epithelium drives a significant change in both gene and miR expression—genes with significant involvement in the inflammatory response as well as in carcinogenesis. The overexpressed miRs (OncomiRs) show a significant effect on the gene expression profile especially for those mRNAs targeted by the oncogenic miRs. These miRs e.g., mmu-miR-155 may further accelerate or cooperate in the development of carcinogenesis by targeting and downregulating genes such as NKX3.1 and Wee1 via their 3′-UTR regions—genes known to enhance the carcinogenic phenotype of the mouse *Pten* model.

Although involved in inflammatory responses, the overexpressed miRs were observed to emanate from the prostate epithelium itself upon in situ hybridisation studies and did not correlate with the location of the immune cell infiltrate. Additionally, miR expression remained high when prostate epithelial cells were grown in culture, indicating immunological cell stimulation, was not responsible. Direct inhibition of the PTEN signalling cascade via temsirolimus treatment reduced miR expression significantly as did ectopic expression of wild type *Pten*. Such inhibitors synergised with miR inhibitors to reduce cell growth, and to re-express several previously downregulated genes.

It is interesting to note that many of the miRs found to be overexpressed in the *Pten* knock out tissue overlap with those found in prostate inflammatory or hyperplastic diseases which are often thought of as being predisposing conditions for PCa and may be strongly linked with its aetiology. For example, miR-155 has been shown to be upregulated in response to bacterial lipopolysaccharides^6,7^. Thus inflammatory pathways driven by PTEN or by a genuine pathogenic responses may have significant overlap—linking inflammation and cancer development.

Synergistic agents based on miR inhibitors may prove worthy in the future as they target the terminal downstream effectors of the overactive PI3k pathway and synergise with agents working upstream, e.g., mTORC or PI3k inhibitors—targeting this pathway from complimentary directions.

## Materials and methods

### Mouse strains

PTen^loxp/loxp^; ARR2-PB-Cre mice, described previously^24^ were originally obtained from The Jackson Laboratory (Maine, USA). All procedures were in accordance with UK Home Office legislation.

### Tissue fixation

Tissues were fixed in 10% formalin for 24 h, then dehydrated in ethanol, xylene and then processed into paraffin embedded blocks, using standard procedures.

### Primary cell culture

Tissue was washed in phosphate-buffered saline (PBS) and macerated using a sterile blade. Tissue was digested in trypsin/collagenase with continued shaking at 37 °C. Cell suspensions were centrifuged at 1200*g* for 5 min and resuspended in medium and plated out in flasks.

### Cell culture

Mouse PTen^−/−^ prostate-derived cells were maintained at 37 °C, 5% CO_2_ in DMEM:Ham’s F12 medium with 10% foetal bovine serum (First Link, Wolverhampton, UK) supplemented with 100units/ml penicillin, 100 mg/ml streptomycin (Sigma, Dorset, UK), pituitary extract (Lonza, Slough, UK), and 10ng/ml epidermal growth factor.

### Immunoblotting and immunohistochemistry

Standard immunoblotting and immunehistochemistry protocols were carried out. Antibodies used were AR (N-20, Santa Cruz, CA, USA). PTen, AKT, p-Akt, NFκβ, IFNγ, HIF1α and STAT3 were obtained from Santa Cruz. CD3 was from DAKO (Santa Clara, CA USA) and CD45R was from Biolegend (San Diego, CA, USA). The Vectastain avidin–biotin complex (Vector Labs, Peterborough, U.K.) was used for detection, using diaminobenzidine chromogenic substrate. Negative controls were included lacking primary antibody. Images were captured using a Leica DM1000 microscope.

### In situ hybridisation

FFPE sections (5 μm), were dewaxed and re-hydrated. Tissue permeabilisation was performed using Protease-K 2–15 μg/ml (Sigma-Aldrich, UK). Slides were washed in diethyl pyrocarbonate (DEPC) water, and incubated in prehybridisation solution for 60 mins at 37 °C, then an equal volume of miR probe (dig-labelled miR155/21/scrambled—250 ng/μl), and incubated overnight. After washing with Tris-buffered saline (TBS), slides were then incubated with anti-digoxigenin alkaline phosphatase antibody (1:600) (Roche) for 60 mins at room temperature. Slides were washed with TBS, then alkaline phosphatase buffer followed by the addition of NBT/BCIP substrate solution (Sigma Aldrich, UK) with 1 μl of 1 μM levamisole and slides were incubated overnight. Finally, slides were washed in water and mounted with aqueous mountant.

### RNA extraction and reverse transcription-polymerase chain reaction (RT-PCR)

Total RNA was prepared using Trizol, (Sigma) and converted to cDNA using the SuperScript First-Strand Synthesis system (ThermoFisher, MA, USA). RNA quality was assessed using a Bioanalyser 2100 (Agilent Technologies LDA, UK).

### Quantitative polymerase chain reaction (qPCR)

Reactions were performed in triplicate in 96-well optical plates on an ABI One-Step system (Applied Biosystems, Warrington, U.K.), consisting of 2 μl cDNA, 7 μl PCR-grade water, 10 μl 2× TaqMan Universal PCR Master Mix (Applied Biosystems), 1 μl Taqman-specific assay probes for RPL19, β-actin, and GAPDH and all microRNAs studied (Applied Biosystems). Parameters were: 50 °C for 2 min, 95 °C for 10 min, 40 cycles of 95 °C for 15 s and 60 °C for 1 min. For *Cre* and *PTen* custom designed oligo primers were purchased (MWG Eurofin, Germany), with SYBR Green PCR. Data were recorded and normalised to GAPDH, β-actin and RPL19.

### MicroRNA RT-qPCR

The rodent Megaplex pool miR-RT stem-looped primers were used for reverse transcription (Applied Biosystems). RNA (500ng) was reverse transcribed using the manufacturer’s conditions. Taqman microRNA microfluidic arrays were used to analyse 715 common rodent microRNAs (A&B Applied Biosystems). Data were normalised using U6-snRNA, snoRNA135, snoRNA202, U87, and Y1.

### Collection and measurement of miRs from mouse blood serum

Blood was collected from Pten^wt^ and Pten^ko^ mice using cardiac puncture under terminal anaesthesia. Blood was collected and was allowed to clot naturally in a sterile Eppendorf tube. Samples were centrifuged at 3000×*g* for 10 min to remove cellular material. The serum was then removed and stored at −80 °C.

For RNA extraction—a standard Trizol LS protocol was carried out (ThermoFisher), with the following modifications. The Trizol LS was ‘spiked’ with *Arabidopsis thaliana* miR-159a and *C. elegans* miR-39 mimic oligos (1 μl/ml of 1 nM solution). 0.75 ml of Trizol LS was added to 0.25 ml of serum for the procedure. During RNA precipitation, 1 μl of GlycoBlue (ThermoFisher) was added to aid visualisation of the RNA pellet. Ten nanograms of RNA was used for the reverse transcription reaction. miRs were detected using microRNA Taqman assays from Life Technologies. MicroRNA levels were the adjusted for any losses using standard curves created using *A. thaliana* and *C. elegans* miR mimic (MWG) in a standard RT reaction.

### RNA-seq analysis

PolyA mRNA was isolated from total RNA using the Dynabeads mRNA DIRECT Kit (Life Technologies, UK). RNA fragment libraries (150–200 bp) were generated using the Ion Total RNA-Seq kit (Life Technologies), ligated to adaptors for cDNA synthesis and amplified using IonXpress RNA-seq barcoded primers (5′). cDNA libraries were clonally amplified using Ion PI template OT2 200 kit (Life technologies, USA) on an Ion OneTouch2 system (Life technologies) as per manufacturer’s instructions. Samples were processed using the Ion Proton 200 sequencing kit and loaded onto a P1 chip and sequenced on an Ion Proton (Life technologies) using default parameters (single-end, forward sequencing). Base calling, adaptor trimming, barcode deconvolution and alignment was performed on Torrent Suite version 3.6 (Life technologies) using the STAR RNA-seq aligner plugin. The Partek Genomic Suite 6.6 software was used for data analysis. The RPKM normalisation method for RNA-seq^69^ was used followed by a 1-way Anova test for differential expression (from *n* = 4 samples per group).

### MiR:mRNA correlation analysis

Genes with a greater ±2-fold change or higher with *p* values of <0.05 or were captured in a database (Access, Microsoft, USA) and merged to the predictive databases MirBase and Targetscan, downloaded from http://www.microrna.org and http://www.targetscan.org/mmu_61 respectively.

### MicroRNA inhibitor transfection

Cells were transfected using RNAiMax (ThermoFisher) with LNA-modified microRNA inhibitors (Exiqon, MA, USA) at 1 μl (of 10 μM stock) per 6-well dish.

### Re-expressing PTen wild type cDNA

The *PTen* coding region was amplified from wild type mouse cDNA using the primers 5′-ATGACAGCCATCATCAAAGAGATCGTTA-3′ and 5′-TCAGACTTTTGTAATTTGTGAATGCTGAT-3′ with *Pfu* polymerase (Promega) and inserted into the pEF6-TOPO expression plasmid (ThermoFisher), and verified by sequencing. The plasmid was transfected into mouse *PTen* ^−/−^ cells using Lipofectamine (ThermoFisher) to generate stable cell lines under blasticidin selection.

### Cloning of miR target genes and 3′-UTR regions and miR binding sites

The coding sequence of Nkx3-1, Wee1 and Fox2 P and their respective 3′-UTR regions were amplified by PCR using *Pfu* and *Taq* polymerase mix (Promega) from mouse cDNA derived from wild type mouse tissue. The primers details are given in Supplemental Table 1. The coding regions were cloned directly into the pEF-TOPO expression plasmid (Life Technologies), and verified by sequencing. The 3′-UTR regions were cloned into the pmiR-Glo luciferase reporter plasmid (Promega) at the *Sac*1/*Sal*1 site of the MCS downstream of the firefly gene *luc*2. For the cloning of miR binding sites we followed the Promega protocol and ligated duplex oligos containing the 22 bp miR binding site, an internal *Not*1 restriction site and 3′*Pme* and 5′*Xba*1 restriction sites.

### Luciferase assays

Mouse cells were transfected with reporter plasmids in a 24-well plate using Lipofectamine 3000 (Life Technologies) and then treated as necessary. After 48 h, cells were washed and lysed in passive lysis buffer (Promega) and the firefly luciferase activity measured using a luminometer. The Promega Dual Glo luciferase assay kit was used to measure both renilla (constitutive) and firefly (variable) activity.

### Biotinylated MiR Mimic Pull Down for mRNA Targets

#### Transfection

Cells were transfected using 200 pmol of biotinylated miR mimic (Sigma) into 4 × 10^6^ cells using Lipofectamine RNAiMax (Life Technologies), and incubated for 24 h. Post transfection, cells were washed with ice-cold PBS and lysed in 1 ml of ice-cold hypotonic lysis buffer (10 mM KCl, 1.5 mM MgCl_2_, 10 mM Tris-HCl pH 7.5, 5 mM Dithiothreitol, 0.5% NP40, 60U/ml SUPERase Inhibitor (Ambion), 5µl/ml protease inhibitors (Roche)). Lysates were centrifuged at 4 °C, 5000×*g* for 5 min and the supernatant collected. Fifty microlitres of supernatant was collected as the input control. NaCl was added to eluates to a final concentration of 1 M.

#### Biotin pull down

A volume of 25 μl of MyOne C1 Dynabeads (LifeTech) was added per sample. Beads had been previously washed with DEPC-treated 0.1 M NaOH, 0.05 M NaCl (twice bead volume), and then washed once in DEPC-treated 0.1 M NaCl and once in hypotonic lysis buffer. Beads were blocked with 1 μg/μl bovine serum albumin and 1 μg/μl yeast tRNA prepared in hypotonic lysis buffer and rotated for 3 h at 4 °C. Samples and beads were incubated for 30 min with rotation and then placed on a magnetic stand and the remove supernatant removed. The beads were washed three times with 200 μl of hypotonic lysis buffer. RNA was extracted from the beads using a standard Trizol extraction, and RNA was reverse transcribed into cDNA for qPCR analysis. Data analysis was compared to % of input controls.

### Drug treatments

Temsirolimus, SF1670, bpV(HOpic) and bicalutamide (Sigma, UK) were dissolved in dimethyl sulfoxide (DMSO; 10 mM stock). These were diluted to 0–100 μM working concentrations in media.

## Electronic supplementary material

Supplemental Table A

Supplemental Table B

Supplemental Table C

Supplemental Table D

Supplemental Table E

Supplemental Table F

Supplemental figure 1

Supplemental figure 2

Supplemental figure 3

Supplemental figure 4

Supplemental figure 5
