# Supplementary material for: Prostate-specific PTen deletion in mice activates inflammatory microRNA expression pathways in the epithelium early in hyperplasia development
Source: Oncogenesis. 2017 Dec 14;6(12):400. doi: 10.1038/s41389-017-0007-5 (PMC5865543; doi:10.1038/s41389-017-0007-5)
Supplement: Supplementary file 1 — Supplemental Table A [file 41389_2017_7_MOESM1_ESM.docx]

| **Lost in PTen KO** | **Expression level** |  | **Expressed in PTen only KO** | **Expression level** |
| --- | --- | --- | --- | --- |
| mmu-miR-615-3p | 7E-10 |  | mmu-miR-139-3p | 1151.3 |
| rno-miR-379* | 1E-5 |  | mmu-miR-217 | 576.0 |
| mmu-miR-599 | 3E-5 |  | rno-miR-346 | 573.2 |
| hsa-miR-144 | 0.0001 |  | mmu-miR-501-3p | 506.5 |
| mmu-miR-370 | 0.0002 |  | mmu-miR-665 | 135.0 |
| mmu-miR-467H | 0.001 |  | mmu-miR-129-3p | 133.8 |
| rno-miR-465 | 0.001 |  | mmu-miR-467c | 120.8 |
| mmu-miR-325 | 0.001 |  | mmu-miR-297b-5p | 107.3 |
| mmu-miR-712* | 0.001 |  | mmu-miR-543 | 102.1 |
| mmu-miR-483 | 0.001 |  | mmu-miR-107 | 92.1 |
| mmu-miR-669D | 0.001 |  | mmu-miR-10b* | 74.0 |
| mmu-miR-466b-3-3p | 0.001 |  | mmu-miR-546 | 65.8 |
| mmu-miR-880 | 0.001 |  | rno-miR-343 | 63.3 |
| mmu-miR-1966 | 0.001 |  | mmu-miR-105 | 62.4 |
| rno-miR-135a* | 0.001 |  | mmu-miR-295* | 54.8 |
| mmu-miR-883B-5P | 0.002 |  | mmu-miR-147 | 53.6 |
| mmu-miR-466a-3p | 0.002 |  | mmu-miR-34c | 49.7 |
| rno-miR-294 | 0.002 |  | rno-miR-351 | 42.9 |
| mmu-miR-543 | 0.002 |  | mmu-miR-504 | 42.5 |
| mmu-miR-715 | 0.003 |  | mmu-let-7c-1* | 41.5 |

Supplemental Table A:

Expression levels of microRNAs either lost or expressed only in PTen^-/-^. Values are compared to the wild type Ct value of 40 cycles.
