# Supplementary material for: Prostate-specific PTen deletion in mice activates inflammatory microRNA expression pathways in the epithelium early in hyperplasia development
Source: Oncogenesis. 2017 Dec 14;6(12):400. doi: 10.1038/s41389-017-0007-5 (PMC5865543; doi:10.1038/s41389-017-0007-5)
Supplement: Supplementary file 2 — Supplemental Table B [file 41389_2017_7_MOESM2_ESM.docx]

| **Gene Name** | **Gene name (generic)** | **Protein function** | **p-value** | **Fold Change** |
| --- | --- | --- | --- | --- |
| Urah | *Urate (5-hydroxyiso-) hydrolase* | Hydrolase which degrades 5-hydroxyisourate. | 2.92E-08 | 13.4679 |
| Fam129b | *Family With Sequence Similarity* 129, *Member* B | May play a role in apoptosis suppression. May promote cell invasion. | 4.15E-08 | 3.29201 |
| Incenp | *Inner Centromere Protein Antigens* 135 | Centromere-interacting proteins, binds directly to microtubules. | 7.10E-08 | 5.55862 |
| Myl12b | *Myosin, Light Chain* 12B, *Regulatory* | Important role in smooth muscle and non-muscle cell contractile activity via its phosphorylation. | 7.31E-08 | 2.32677 |
| Dok1 | *Docking Protein* 1 | Transduction pathway downstream of receptor tyrosine kinases - a scaffold protein that helps the assembly of multiprotein signalling complexes. | 8.49E-08 | 7.24569 |
| Arpc1b | *Actin Related Protein* 2/3 *Complex* | Control of actin polymerization in cells. | 8.65E-08 | 3.20972 |
| Wnt4 | *Wingless-Type MMTV Integration Site Family* | Secreted signaling proteins. These proteins have been implicated in oncogenesis and in several developmental processes. | 9.15E-08 | 32.5412 |
| Ppap2c | *Phosphatidic Acid Phosphatase Type 2C* | Converts phosphatidic acid to diacylglycerol | 9.78E-08 | 3.08595 |
| Hacd3 | 3-*Hydroxyacyl-CoA Dehydratase* 3 | Catalyzes the third of the four reactions of the long-chain fatty acids elongation cycle. | 2.35E-07 | 4.43229 |
| Epsti1 | *Epithelial Stromal Interaction* 1 | Stromal fibroblast-induced gene. Involved in metastasis and invasion. | 2.41E-07 | 13.8527 |
| Traf3 | *TNF Receptor-Associated Factor* 3 | Member of the TNF receptor associated factor (TRAF) protein family. TRAF proteins associate with, and mediate the signal transduction from, members of the TNF receptor. | 2.73E-07 | 3.04191 |
| Baz1a | *Bromodomain Adjacent To Zinc Finger Domain,* 1A | ATP-dependent chromatin assembly factor (ACF). | 2.74E-07 | 2.26297 |
| Mdfi | *MyoD Family Inhibitor* | Transcription factor that negatively regulates other myogenic family proteins. | 2.88E-07 | 28.4105 |
| Ctse | *Cathepsin* E | Aspartyl protease – roles in antigenic peptides presentation, and on epithelial mucus-producing cells. Roles in cancer progression. | 3.06E-07 | 61.9217 |
| Cxcl16 | *Chemokine (C-X-C Motif) Ligand* 16 | Scavenger receptor on macrophages, which specifically binds to OxLDL (oxidized low density lipoprotein. | 3.67E-07 | 10.9309 |
| Sh3pxd2a | *SH3 And PX Domains* 2A | Adapter protein involved in invadopodia and podosome formation, extracellular matrix degradation and invasiveness of some cancer cells. Binds matrix metalloproteinases. Phosphatidylinositol binding. | 3.80E-07 | 8.81136 |
| Tbl1xr1 | *Transducin (Beta)-Like 1 X-Linked Receptor* 1 | F-box-like, involved in ubiquitin/19S recruitment to nuclear receptor-regulated transcription units. Essential in transcription activation mediated by nuclear receptors. | 4.58E-07 | 3.54353 |
| Nek6 | *NIMA-Related Kinase* 6 | Kinase required for progression through the metaphase portion of mitosis. | 5.08E-07 | 15.8877 |
| Cldn7 | *Claudin* 7 | Integral membrane proteins and components of tight junction strands. | 5.64E-07 | 2.53088 |
| Mbd4 | *Methyl-CpG Binding Domain Protein* 4 | These proteins are capable of binding specifically to methylated DNA, and some members can also repress transcription from methylated gene promoters | 5.94E-07 | 2.95162 |

Supplemental Table B: List of the top 20 upregulated genes (Refseq 2015 nomenclature) in PTen^-/-^ mouse prostate tissue, ranked according to lowest P value. Table also lists their fold change value for comparison.
