# Supplementary material for: Prostate-specific PTen deletion in mice activates inflammatory microRNA expression pathways in the epithelium early in hyperplasia development
Source: Oncogenesis. 2017 Dec 14;6(12):400. doi: 10.1038/s41389-017-0007-5 (PMC5865543; doi:10.1038/s41389-017-0007-5)
Supplement: Supplementary file 3 — Supplemental Table C [file 41389_2017_7_MOESM3_ESM.docx]

| **Gene Name** | **Gene name (generic)** | **Protein function** | **p-value** | **Fold Change** |
| --- | --- | --- | --- | --- |
| Ear2 | *Eosinophil-associated, ribonuclease A* | Nucleic acid binding and ribonuclease activity. | 0.00800558 | 1.59E+11 |
| Cyp4f40 | *Cytochrome P450, Family 4* | Monooxygenases - catalyzes many reactions involved in drug metabolism and synthesis of cholesterol, steroids and other lipids. | 0.0413034 | 1.21E+09 |
| Cntnap5b | *Contactin Associated Protein-Like* 5 | Cell adhesion molecules and receptors. | 0.00934505 | 8.45E+06 |
| Cyp4a31 | Cytochrome P450, Family 4 | Monooxygenases - catalyzes many reactions involved in drug metabolism and synthesis of cholesterol, steroids and other lipids. | 0.0270051 | 120998 |
| Lgals6 | *Lectin, Galactoside-Binding, Soluble*, 4 | Beta-galactoside-binding proteins implicated in modulating cell-cell and cell-matrix interactions. | 0.000943184 | 105225 |
| Reg3b | *Regenerating Islet-Derived 3 Alpha* | Involved in cell proliferation or differentiation. | 0.0465751 | 7157.95 |
| Tff3 | *Trefoil Factor* 3 | Regulates cell migration, invasion, and angiogenesis, enhances ERG-mediated cell invasion in CRPC prostate cancer cells. | 0.000279639 | 5249.95 |
| Klk1 | *Kallikrein* 1 | Serine proteases may produce vasoactive peptides. | 0.00501787 | 4784.18 |
| Reg1 | *Regenerating Islet-Derived 1 Alpha* | Involved in cell proliferation or differentiation. | 0.046921 | 3988.56 |
| Wfdc2 | *WAP Four-Disulfide Core Domain* 2 | Protease inhibitor. | 0.0369315 | 2452.37 |
| Gp9 | *Glycoprotein* IX | Platelet surface membrane glycoprotein. | 0.0103189 | 2348.3 |
| Wfdc18 | WAP Four-Disulfide Core Domain | Protease inhibitor. | 0.00148439 | 2218.93 |
| Gsdmc3 | *Gasdermin* C | Expressed on mucus producing cells, associated with metastasis. | 0.0276458 | 1838.75 |
| Ugt2b34 | *UDP glucuronosyltransferase* 2 | Importance in the conjugation and subsequent elimination of potentially toxic xenobiotics. | 0.0183935 | 1476.48 |
| Gsdmc2 | *Gasdermin* C | Expressed on mucus producing cells, associated with metastasis. | 0.0209716 | 1381.51 |
| Sval1 | Seminal vesicle antigen-like 1 | Present specifically in the seminal plasma. | 0.0180678 | 1163.23 |
| Reg3g | *Regenerating Islet-Derived 3 Alpha* | Involved in cell proliferation or differentiation. | 0.0166814 | 1135.45 |
| Gsdmc4 | Gasdermin C | Expressed on mucus producing cells, associated with metastasis. | 0.0100277 | 1071.06 |
| Gsdmc | Gasdermin C | Expressed on mucus producing cells, associated with metastasis. | 0.0155207 | 933.368 |
| Sprr2a3 | Small proline-rich protein 2A3 | Envelope protein of keratinocytes. | 0.00970359 | 922.092 |

Supplemental Table C: List of the top 20 upregulated genes (Refseq 2015 nomenclature) in PTen^-/-^ mouse prostate tissue, ranked according to fold change. Table also lists their P values value for comparison.
