# Supplementary material for: Prostate-specific PTen deletion in mice activates inflammatory microRNA expression pathways in the epithelium early in hyperplasia development
Source: Oncogenesis. 2017 Dec 14;6(12):400. doi: 10.1038/s41389-017-0007-5 (PMC5865543; doi:10.1038/s41389-017-0007-5)
Supplement: Supplementary file 4 — Supplemental Table D [file 41389_2017_7_MOESM4_ESM.docx]

| **Gene Name** | **Gene name (generic)** | **Protein function** | **p-value** | **Fold Change** |
| --- | --- | --- | --- | --- |
| Chn2 | *Chimerin2* | Translocates from the cytosol to the Golgi apparatus membrane upon binding by diacylglycerol - important in cell proliferation and migration. | 1.77E-09 | -137.678 |
| Bmp7 | *Bone Morphogenetic Protein 7* | Family of secreted signaling molecules that can induce ectopic bone growth - part of the transforming growth factor-beta (TGFB) superfamily. | 1.21E-08 | -5.49778 |
| Extl1 | *Exostosin-Like Glycosyltransferase 1* | 1,4- N-acetylglucosaminyltransferase involved in chain elongation of heparan sulfate. | 2.54E-08 | -4.76253 |
| Tmod4 | *Tropomodulin* 4 | Actin binding and tropomyosin binding | 3.75E-08 | -18.6985 |
| Macrod1 | *MACRO Domain Containing 1* | O-acetyl-ADP ribose deacetylase, important role in ER and AR signalling, amplifies the transactivation function of AR in response to androgen. | 1.47E-07 | -4.58676 |
| Apoa5 | *Apolipoprotein* A-V | Apolipoprotein - regulates triglyceride levels. | 2.00E-07 | -12.0369 |
| Skida1 | *SKI/DACH Domain Containing 1* | Nucleotide binding. | 2.31E-07 | -2.87951 |
| Gad2 | *Glutamate* *Decarboxylase* 2 | Glutamic acid decarboxylase. | 2.37E-07 | -58.5537 |
| Atp6v1c2 | *ATPase, H+ Transporting, Lysosomal 42kDa, V1 Subunit* C2 | Vacuolar ATPase. | 2.52E-07 | -20.808 |
| Arid3c | *AT Rich Interactive Domain* 3C | Helix-turn-helix DNA-binding domain - roles in embryonic patterning, cell lineage gene regulation, cell cycle control, transcriptional regulation and chromatin structure modification. | 3.56E-07 | -47.0205 |
| Vnn1 | *Vanin* 1 | Membrane-associated proteins, role in hematopoietic cells may play a role in oxidative-stress response. | 4.78E-07 | -14.042 |
| Cds2 | *CDP-Diacylglycerol Synthase* | Regulates the amount of phosphatidylinositol available for signalling. | 5.14E-07 | -2.40204 |
| Gars | *Glycyl-TRNA Synthetase* | Aminoacyl-tRNA synthetases. | 6.38E-07 | -5.00176 |
| Itpk1 | *Inositol-Tetrakisphosphate 1-Kinase* | Phosphorylate various inositol polyphosphate such as Ins(3,4,5,6)P4 or Ins(1,3,4)P3 | 7.34E-07 | -5.90285 |
| Lysmd1 | *LysM, Putative Peptidoglycan-Binding, Domain Containing* 1 | Peptidoglycan-Binding. | 8.34E-07 | -20.3813 |
| Me2 | Malic Enzyme 2, NAD(+)-Dependent | Mitochondrial NAD-dependent malic enzyme. | 1.00E-06 | -5.37482 |
| Tbc1d9 | *TBC1 Domain Family, Member* 9 | GTPase-activating protein for Rab. | 1.03E-06 | -4.38791 |
| Lars | *Leucyl-TRNA Synthetase* | Aminoacyl-tRNA synthetase family. | 1.10E-06 | -2.16261 |
| Farsb | *Phenylalanyl-TRNA Synthetase* | Aminoacyl-tRNA synthetase family. | 1.21E-06 | -2.43575 |
| Slc30a2 | *Solute Carrier Family 30* | Zinc Transporter | 1.58E-06 | -87.453 |

Supplemental Table D:

List of the top 20 downregulated genes (Refseq 2015 nomenclature) in PTen^-/-^ mouse prostate tissue, ranked according to lowest P value. Table also lists their fold change value for comparison.
