# Supplementary material for: Prostate-specific PTen deletion in mice activates inflammatory microRNA expression pathways in the epithelium early in hyperplasia development
Source: Oncogenesis. 2017 Dec 14;6(12):400. doi: 10.1038/s41389-017-0007-5 (PMC5865543; doi:10.1038/s41389-017-0007-5)
Supplement: Supplementary file 5 — Supplemental Table E [file 41389_2017_7_MOESM5_ESM.docx]

| **Gene Name** | ***Gene name (generic)*** | **Protein function** | **p-value** | **Fold Change** |
| --- | --- | --- | --- | --- |
| Ren2 | *Renin* | Catalyzes the activation pathway of angiotensinogen--a cascade. | 0.000229098 | -11532.7 |
| Ren1 | *Renin* | Catalyzes the activation pathway of angiotensinogen--a cascade. | 2.73E-05 | -10593.7 |
| Col4a6 | *Collagen, Type IV, Alpha 6* | One of the six subunits of type IV collagen, the major structural component of basement membranes. | 0.000349267 | -939.023 |
| Glb1l3 | *Galactosidase, Beta 1-Like 3* | Beta-galactosidase activity. | 3.48E-05 | -848.568 |
| Tgm4 | *Transglutaminase 4* | Catalyzes the cross-linking of proteins polyamines to specific proteins in the seminal tract. | 9.01E-06 | -760.59 |
| C1rb | *Complement Component 1, R Subcomponent* | Serine protease, -the first component of the classical complement system. | 1.62E-05 | -663.375 |
| Pnliprp1 | *Pancreatic Lipase-Related Protein 1* | Inhibitor of dietary triglyceride digestion. | 0.000198595 | -605.998 |
| Gsdma3 | *Gasdermin A* | Expressed in mucus secreting cells. | 4.26E-06 | -327.741 |
| Serpina5 | *Serpin Peptidase Inhibitor* | Inhibitors of serine proteases, including protein C and various plasminogen activators and seminal fluid kallikreins. | 0.00490805 | -287.954 |
| Mt3 | *Metallothionein 3* | Binds heavy metals. | 0.000312562 | -240.784 |
| Gsdma | *Gasdermin A* | Expressed in mucus secreting cells. | 1.67E-06 | -213.65 |
| Pbsn | *Probasin* | Carrier of hydrophobic ligands in ejaculate. | 0.000313794 | -198.602 |
| C1s2 | *Complement component 1* | Compliment system component | 6.09E-05 | -197.54 |
| Fgl1 | *Fibrinogen-Like 1* | Homologous to the fibrinogen-type proteins. | 0.000244523 | -197.356 |
| Olfr829 | *Olfactory receptor 829* | Receptors that interact with odorant molecules. | 0.00522923 | -192.439 |
| Gpha2 | *Glycoprotein Hormone Alpha 2* | Subunit of the dimeric glycoprotein hormone family. | 2.80E-05 | -185.787 |
| Chn2 | *Chimerin 2* | Translocates from the cytosol to the Golgi apparatus membrane upon binding by diacylglycerol - important in cell proliferation and migration. | 1.77E-09 | -137.678 |
| Kiss1 | *KiSS-1 Metastasis-Suppressor* | Inhibit chemotaxis and invasion. | 0.00242003 | -126.485 |
| Gsdma2 | *Gasdermin A* | Expressed in mucus secreting cells. | 0.000369638 | -120.144 |
| Nkx3-1 | *NK3 Homeobox 1* | Homeobox-containing transcription factor - functions as a negative regulator of epithelial cell growth in prostate tissue | 0.000243903 | -106.943 |

Supplemental Table E:

List of the top 20 downregulated genes (Refseq 2015 nomenclature) in PTen^-/-^ mouse prostate tissue, ranked according to fold change. Table also lists their P values for comparison.
