# Supplementary material for: Prostate-specific PTen deletion in mice activates inflammatory microRNA expression pathways in the epithelium early in hyperplasia development
Source: Oncogenesis. 2017 Dec 14;6(12):400. doi: 10.1038/s41389-017-0007-5 (PMC5865543; doi:10.1038/s41389-017-0007-5)
Supplement: Supplementary file 6 — Supplemental Table F [file 41389_2017_7_MOESM6_ESM.docx]

**Primers for cloning the coding sequence (5’ – 3’)**

Wee1 for ATGAGCTTCCTGAGCCGACA

Wee1 rev TCAGTATATAGTAAGGCTGACAGAG

Fox2p for ATGATGCAGGAATCTGCGACAGA

Fox2p rev TCATTCCAGGTCCTCAGATAAAG

NKX3-1 For atgcttagggtagcggag

NKX3-1 Rev ctaccagaaagatggatg

**Primers for cloning the 3’ UTR (5’ – 3’)**

Nkx3.1 for [GAGCTC]CAACAGTTCTGCTGATAACTGCAT

Nkx3.1 rev [GTCGAC]GAGTACTATACTAAATGCCAAGTGAC

Wee1 For [GAGCTC]ACTGCTCACATTCCCCAGC

Wee1 Rev [GTCGAC]AAGATTTAGACAATTAAGGTAAGCACGG

Foxp2 for [CTCGAG]GAACGAACTTGTGACACCTCAGT

Foxp2 rev [GTCGAC]AAATGGGTCAAAAAGACTTAGATCTTTTTTTATTTG
