# Supplementary material for: Prostate-specific PTen deletion in mice activates inflammatory microRNA expression pathways in the epithelium early in hyperplasia development
Source: Oncogenesis. 2017 Dec 14;6(12):400. doi: 10.1038/s41389-017-0007-5 (PMC5865543; doi:10.1038/s41389-017-0007-5)
Supplement: Supplementary file 7 — Supplemental figure 1 [file 41389_2017_7_MOESM7_ESM.pdf]

Supplemental figure 1.

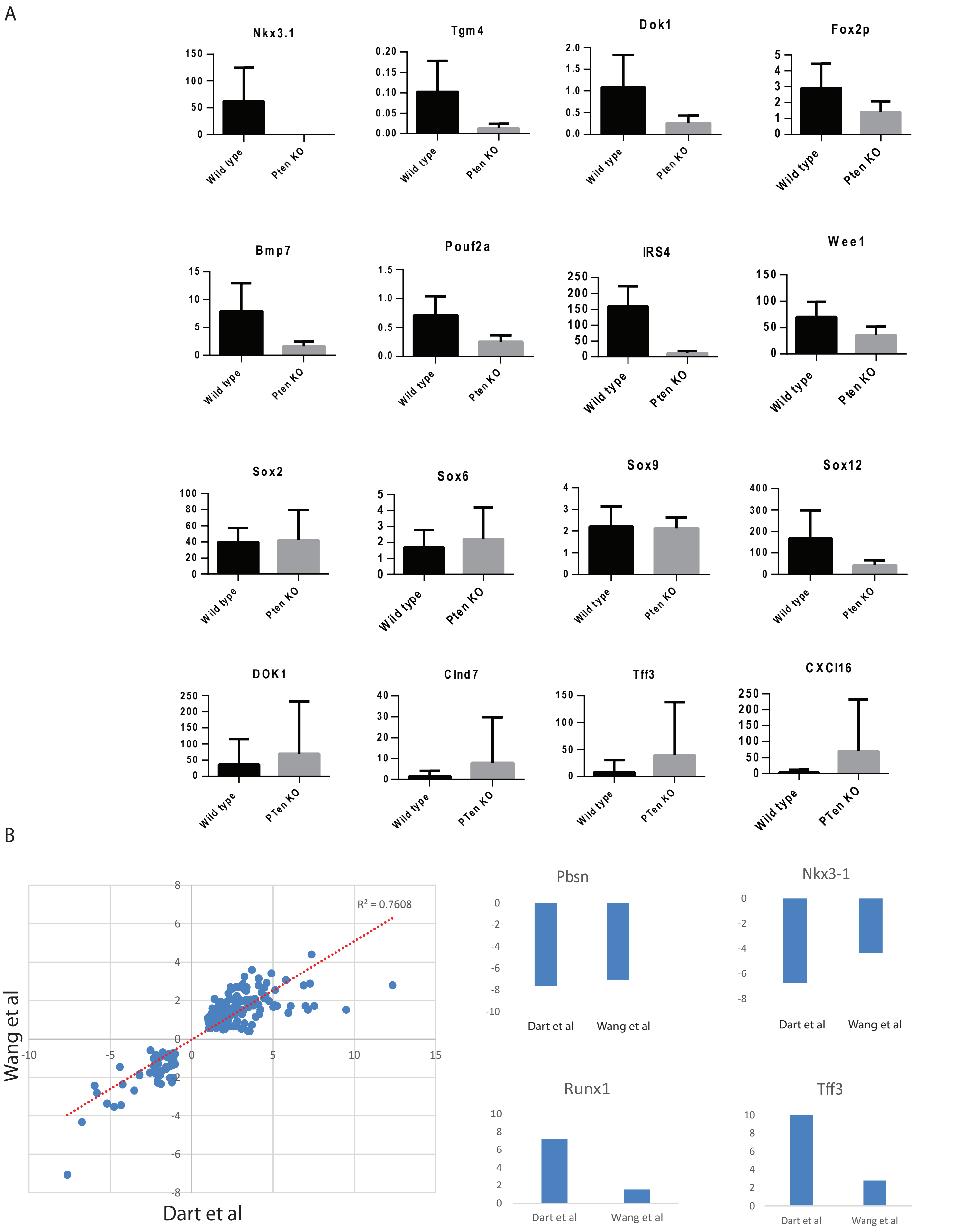

A, Q-PCR validation of a selection of target genes changed in the PTen-/- mose prostate compared to wild type. Data represents N=12 samples in each group. B, Comparison of gene array data from Wang et al, versus RNA-seq data (Dart et al) for the same gene set. Left hand side shows a correlation plot for the entire gene set. Right hand side shows examples of the highest expressed or lowest expressed genes in the PTen -/- sample sets.
