# Supplementary material for: Prostate-specific PTen deletion in mice activates inflammatory microRNA expression pathways in the epithelium early in hyperplasia development
Source: Oncogenesis. 2017 Dec 14;6(12):400. doi: 10.1038/s41389-017-0007-5 (PMC5865543; doi:10.1038/s41389-017-0007-5)
Supplement: Supplementary file 8 — Supplemental figure 2 [file 41389_2017_7_MOESM8_ESM.pdf]

Supplemental figure 2

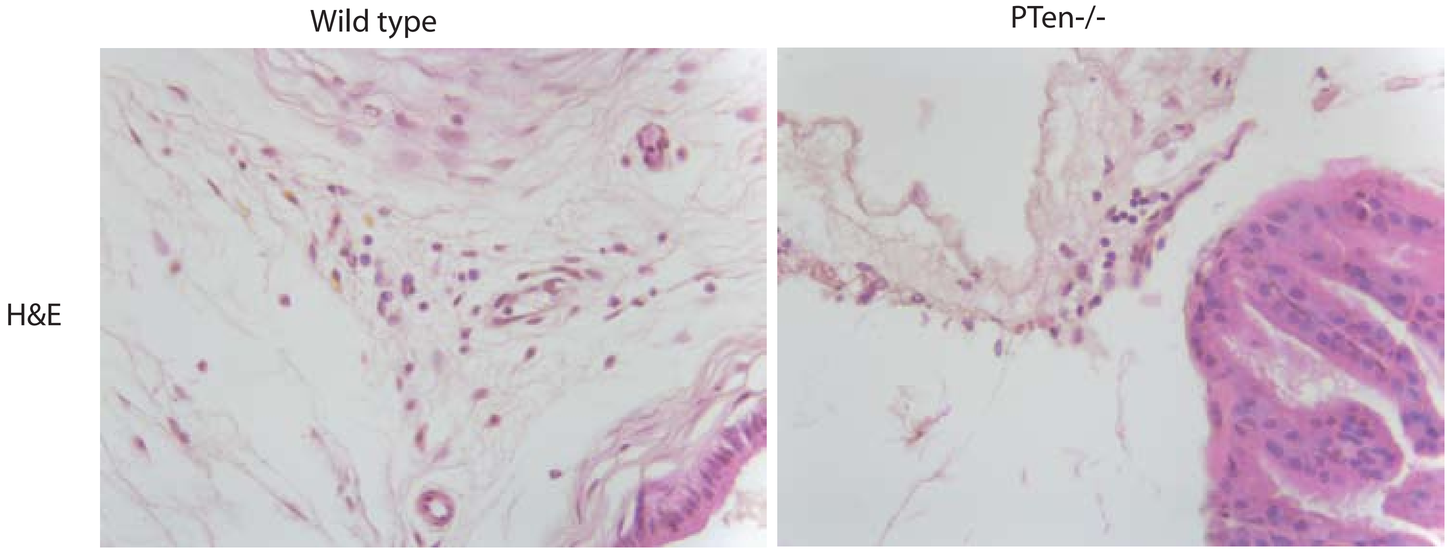

Hematoxillin and eosin staining of mouse prostate tissue - visually analysed for immune cell infiltrate.
