# Supplementary material for: Prostate-specific PTen deletion in mice activates inflammatory microRNA expression pathways in the epithelium early in hyperplasia development
Source: Oncogenesis. 2017 Dec 14;6(12):400. doi: 10.1038/s41389-017-0007-5 (PMC5865543; doi:10.1038/s41389-017-0007-5)
Supplement: Supplementary file 9 — Supplemental figure 3 [file 41389_2017_7_MOESM9_ESM.pdf]

Supplemental figure 3.

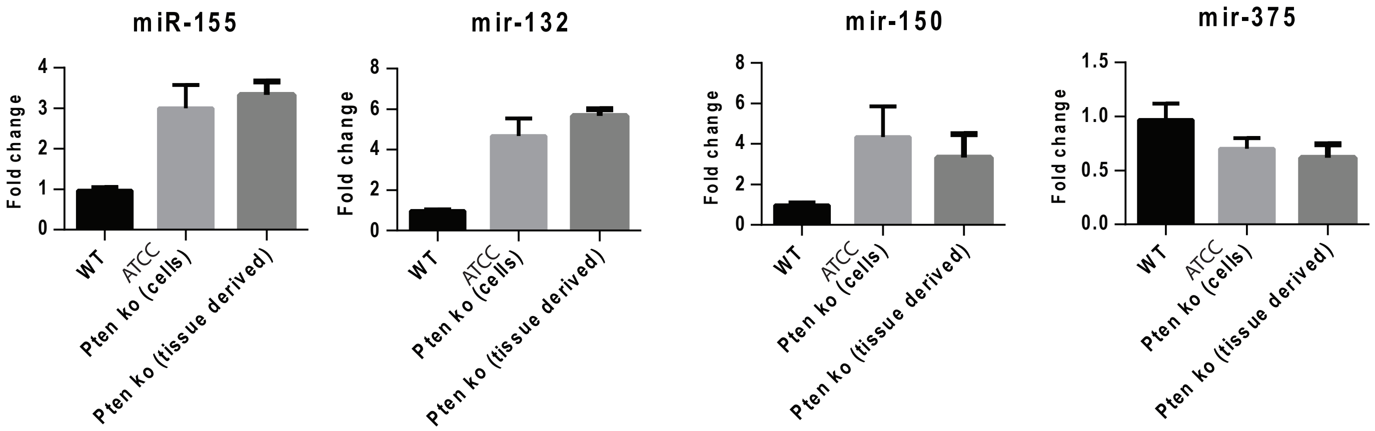

Q-PCR analysis of mir-155, 132, 150 and 375 levels in PTen kockout cells derived from the ATCC or derived from our 'in house' mouse PTen deletion models. Levels are compared with wild type mouse tissue for comparison. Data is normalised to the sno-RNAs 202 & 234.
