# Supplementary material for: Prostate-specific PTen deletion in mice activates inflammatory microRNA expression pathways in the epithelium early in hyperplasia development
Source: Oncogenesis. 2017 Dec 14;6(12):400. doi: 10.1038/s41389-017-0007-5 (PMC5865543; doi:10.1038/s41389-017-0007-5)
Supplement: Supplementary file 10 — Supplemental figure 4 [file 41389_2017_7_MOESM10_ESM.pdf]

Supplemental figure 4

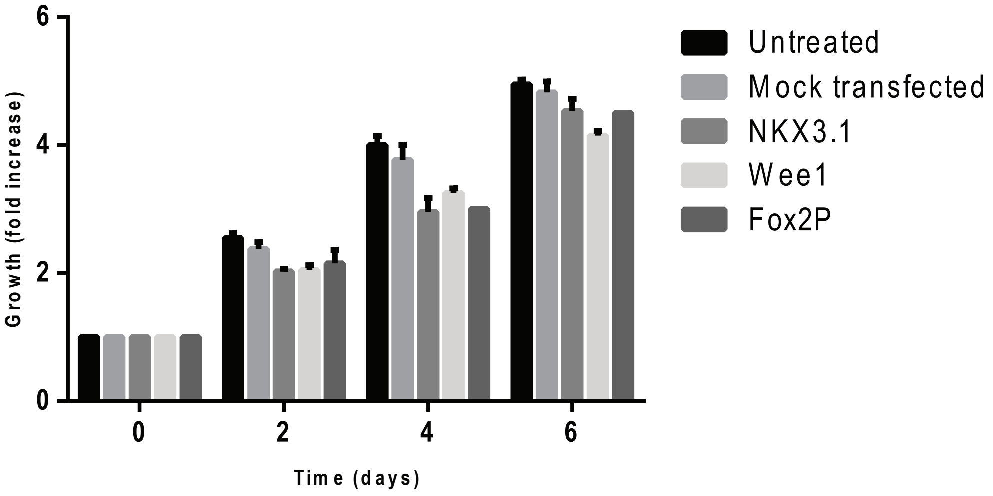

Crystal violet growth assays of PTen-/- cells over 6 days after transfection with pEF6-NKX3.1, Wee1 and Fox2P expressing plasmids. Data represents the mean of three replicates and standard error.
