# Supplementary material for: Prostate-specific PTen deletion in mice activates inflammatory microRNA expression pathways in the epithelium early in hyperplasia development
Source: Oncogenesis. 2017 Dec 14;6(12):400. doi: 10.1038/s41389-017-0007-5 (PMC5865543; doi:10.1038/s41389-017-0007-5)
Supplement: Supplementary file 11 — Supplemental figure 5 [file 41389_2017_7_MOESM11_ESM.pdf]

Supplemental figure 5.

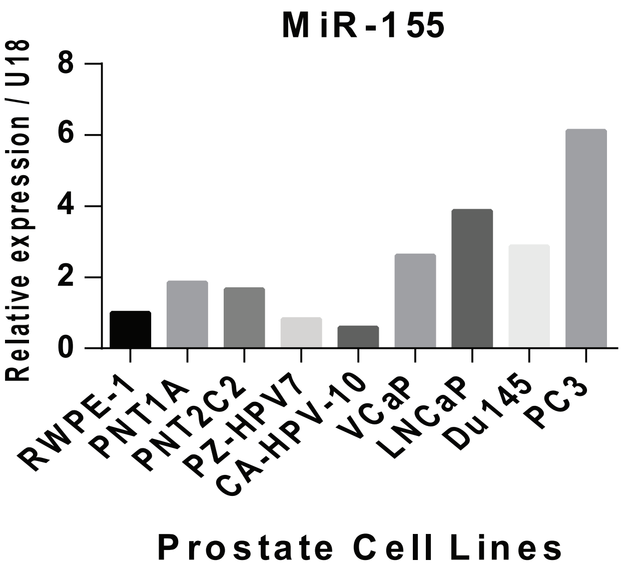

Q-PCR analysis of miR-155 in a panel of human cell lines, representing those derived from more normal prostate tissue on the left leading to more aggressive or metastasis-derived cell lines on the right. Data represents miR levels normalised to U18, and then expressed as fold change over RWPE-1.
